# Choice of antithrombotic therapy for patients with atrial fibrillation undergoing carotid angioplasty and stenting: a nationwide population-based study

**DOI:** 10.1038/s41598-022-05546-8

**Published:** 2022-01-26

**Authors:** Yung-Chuan Huang, Yen-Chun Huang, Yu-Chen Cheng, Mingchih Chen

**Affiliations:** 1 Department of Neurology, Fu Jen Catholic University Hospital, Fu Jen Catholic University, No. 69, Guizi Rd., Taishan Dist., New Taipei City, 24352 Taiwan (R.O.C.); 2 Graduate Institute of Business Administration, College of Management, Fu Jen Catholic University, New Taipei City, Taiwan; 3 Artificial Intelligence Development Center, Fu Jen Catholic University, New Taipei City, Taiwan

**Keywords:** Stroke, Atrial fibrillation, Preventive medicine

## Abstract

Nonvalvular atrial fibrillation (NVAF) and carotid stenosis are important risk factors for stroke. Carotid angioplasty and stent placement (CAS) is recommended for patients with symptomatic high-grade carotid stenosis. The optimal medical management for patients with NVAF after CAS remains unclear. We aimed to clarify this issue using real-world data from the Taiwanese National Health Insurance Research Database (NHIRD). In total, 2116 consecutive NVAF patients who received CAS between January 1, 2010, and December 31, 2016, from NHIRD were divided into groups based on post-procedure medication as follows: only antiplatelet agent (OAP, n = 587); only anticoagulation agent (OAC, n = 477); dual antiplatelet agents (DAP, n = 49); and a combination of antiplatelet and anticoagulation agents (CAPAC, n = 304). Mortality, vascular events, and major bleeding episodes were compared after matching with the Charlson comorbidity index and CHA2DS2-VASc score. The CAPAC and the OAC groups had lower mortality rates than the OAP group (*P* = 0.0219), with no statistical differences in major bleeding, ischemic stroke, or vascular events. Conclusively, OAC therapy after CAS appears suitable for NVAF patients. CAPAC therapy might be considered as initial therapy or when there is concern about vascular events.

## Introduction

Nonvalvular atrial fibrillation (NVAF) is the most common risk factor for cardiogenic stroke, with a global prevalence of approximately 1–2%^1^. Thrombus formation in the left atrial appendage causes cerebral and systemic embolism^2^. In Asia, the stroke burden of atrial fibrillation (AF) patients in community-based cohorts was estimated to be 13–15% per year^3^. According to a cohort study using data from the National Health Insurance Research Database (NHIRD), Taiwanese have a high annual stroke rate exceeding 5% when the CHA2DS2-VASc (congestive heart failure, hypertension, age 75 years or older, diabetes mellitus, previous stroke or transient ischemic attack, vascular disease, age 65–74 years, female sex) score is greater than 4^4^. Warfarin was the standard treatment for these patients. Recently, non-vitamin K antagonist oral anticoagulants (NOACs) such as direct thrombin inhibitors (dabigatran) and factor Xa inhibitors have provided effective and safe protection^5–8^. However, some patients with AF and carotid stenosis have a significantly higher stroke recurrence rate than those with only AF^9^. The rupture of atherosclerotic plaques, which have a lipid core surrounded by fibrous tissue^10^, may activate platelet adhesion and aggregation, blocking blood flow. Patients with severe carotid atherosclerosis have a high risk of coronary heart disease and sudden death^11^. Therefore, antiplatelet agents such as aspirin and P2Y12 inhibitors that inhibit platelet aggregation are the best choice for carotid atherosclerosis.

Carotid atherosclerosis with high-grade stenosis coexisting with NVAF is common with a 5–8% frequency in the aging population. The conditions have similar risk factors^12,13^. AF was recorded in more than 10% of patients who received interventions for carotid stenosis^14–16^. Concomitant AF was an independent risk factor for long-term stroke recurrence, mortality, and perioperative adverse events in these studies. For patients with severe symptomatic carotid stenosis, carotid endarterectomy or carotid angioplasty and stent placement (CAS) are the best management choices to reduce the risk of stroke^17^. The current consensus for treatment to prevent restenosis and vascular events after CAS is antiplatelet drugs or even dual antiplatelet drugs plus statins, with improved risk factor control.

Antiplatelet and anticoagulation agents seem equally necessary for patients with AF and severe carotid stenosis after vascular intervention. A combination of anticoagulation and dual antiplatelet agents appears to be a reasonable choice; however, there are concerns about a high bleeding risk^18^. There is recent preliminary evidence concerning a similar dilemma in the choice of medical therapy after acute coronary syndrome or percutaneous coronary intervention (PCI) in patients with AF^19–23^. Combination therapy with one NOAC and a P2Y12 inhibitor provided adequate protection with few bleeding events in these patients^19^. However, there are few studies focusing on antithrombotic agents after CAS, especially in NVAF patients. We aimed to conduct a preliminary evaluation of the effects of different medical therapies for this situation in real-world data.

## Methods

### Data source

This retrospective cohort study used claims data from the NHIRD in Taiwan. The National Health Insurance (NHI) program, which commenced on March 1, 1995, has reimbursed the health care costs of 99.9% of Taiwan’s population since 2014. The NHI program stores detailed health data, including demographic data on the insured population, consultations, diagnostic codes, and drug prescriptions. The International Classification of Diseases, Ninth Edition, Clinical Modification (ICD-9-CM) was used for recording diagnoses in NHIRD until 2016, and the Tenth Edition (ICD-10) has been used after that. Several scientific studies have used this database with high reliability. NHIRD provides a variety of personal medical information and comprehensive long-term follow-up.

### Ethics statement

This research was approved by a full ethical review from Fu Jen Catholic University Institutional Review Board in Taiwan (Approval number: C104016), and all methods were carried out in accordance with relevant guidelines and regulations. The requirement for informed consent was waived by the Fu Jen Catholic University Institutional Review Board in Taiwan because of the retrospective nature of the study and all NHIRD data had been de-identified.

### Study population

**Figure 1:**
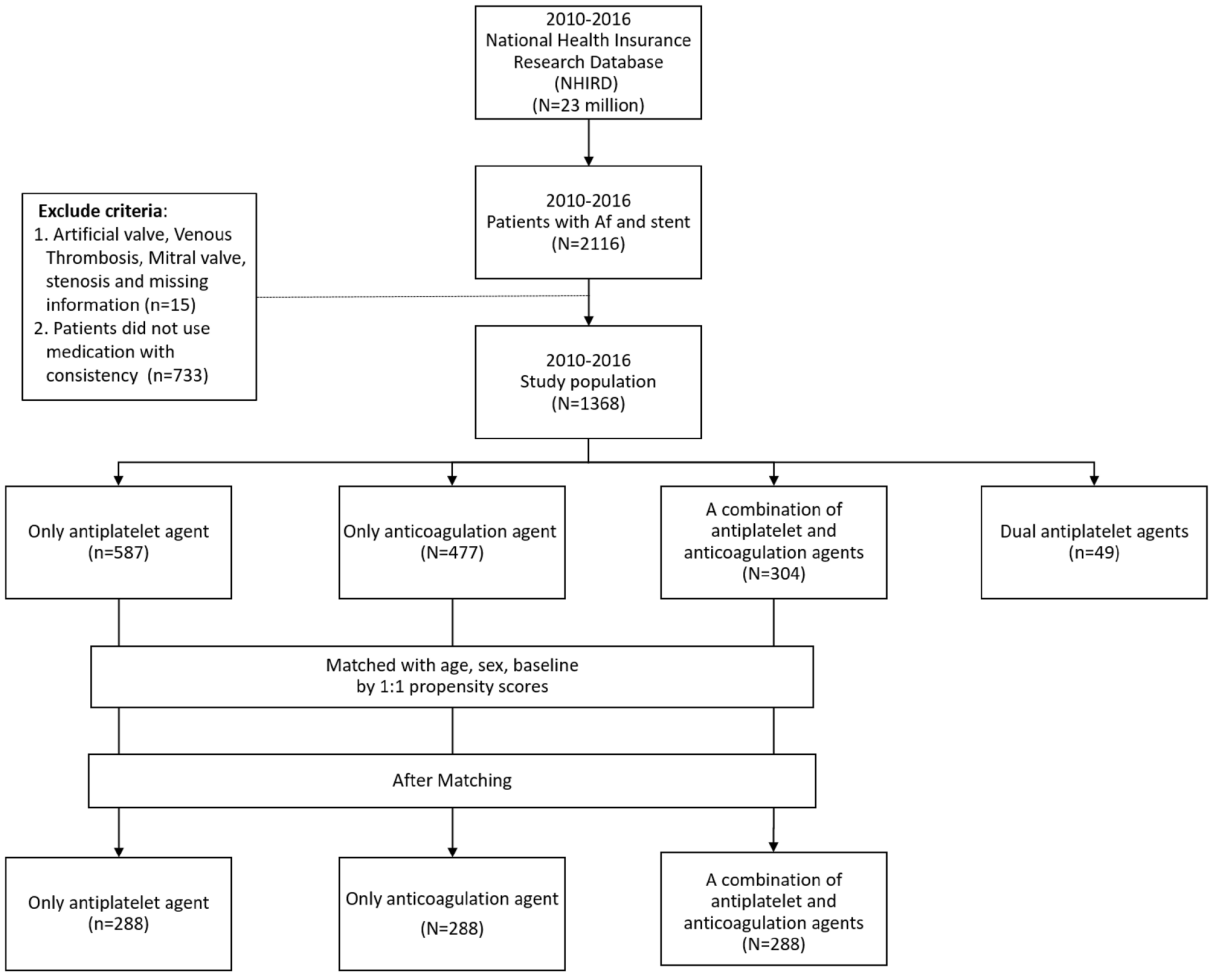
Study flow diagram. We enrolled 2116 consecutive non-valvular atrial fibrillation patients who had successful carotid angioplasty and stent placement, with regular follow-up and consistent drug coherence, from January 1, 2010, to December 31, 2016. They were divided into groups according to treatment. We matched different therapy groups mainly according to age, sex and CHA2DS2-VASc scores for comparison of primary outcomes.

We enrolled consecutive patients diagnosed with AF (ICD-9-CM code 427.31, between January 1, 2010, and December 31, 2015, or ICD-10-CM code I48, between January 1 and December 31, 2016), who received a carotid stent (operation code 33074B and 33128B) and were discharged without complications (in the absence of ICD-9-CM: 996.4 or ICD-10-CM: T82), according to medical records. Patients with the history of prosthetic valve (ICD-9-CM code V43.3, or ICD-10-CM codes Z95.2), venous thrombosis (ICD-9-CM code 453 or ICD-10-CM code I82), mitral valve stenosis (ICD-9-CM code 396 or ICD-10-CM codes I08) were excluded. Further exclusions included patients who did not take any antithrombotic agent or with missing data. Secondary prevention therapy was required for at least 3 months in the 6 months following the vascular intervention; otherwise, patients with poor adherence were excluded. The patients were divided into four groups according to prescribed medicines: only antiplatelet agent (OAP) group, treated with aspirin or clopidogrel; only anticoagulation agent (OAC) group, treated with Warfarin or a NOAC, dual antiplatelet agent (DAP) group, who had aspirin plus clopidogrel and combined antiplatelet and anticoagulation agent (CAPAC) group, who were prescribed an antiplatelet agent plus an anticoagulation agent. The index date was defined as the day they received the first dose of prescribed medicines. (Fig. 1).

### Comorbidities and variable definitions

The baseline characteristics included sex, age, Charlson comorbidity index, and CHA2DS2-VASc scores (congestive heart failure, hypertension, age 75 years or older, diabetes mellitus (DM), previous stroke or transient ischemic attack (TIA), vascular disease, age 65–74 years, female sex), and comorbidities including peripheral vascular disease (PVD), ischemic stroke or systemic embolism, hypertension (HTN), hyperlipidemia, DM, chronic liver disease, chronic kidney disease (CKD), chronic obstructive pulmonary disease (COPD), congestive heart failure (CHF) and TIA. The Charlson comorbidity index score (CCIs) is the classic index in the literature^24^. The comorbidities are weighted from 1 to 6, and a score that reflects long-term outcomes is calculated for patients. Patients with a myocardial infarction (MI), CHF, cerebrovascular disease, connective tissue disease, PVD, dementia, COPD, DM, ulcer disease, or mild liver disease are assigned one point. Hemiplegia, moderate-to-severe renal disease, diabetes-with-end-organ-damage, and any malignancy, including lymphoma and leukemia, except malignant neoplasm of the skin get two points. Three points are given for moderate or severe liver disease, while metastatic solid tumors and acquired immune deficiency syndrome are given six points.

### Definition of outcomes

The primary outcome was all-cause mortality at the 1-year, 2-year, and 3-year follow-up. The secondary outcomes were vascular events, including ischemic stroke and acute MI happening within one year. We also evaluated the incidence of symptomatic hemorrhage within one year and the mortality rate over the whole follow-up period. Patients were followed up until death, vascular event, major bleeding, or the end of the study, whichever came earliest. ICD codes used to define comorbidities and clinical outcomes in our study are listed in Table 1.

**Table 1:** ICD codes used to define comorbidities and clinical outcomes.

Disease	ICD-9 Codes	ICD-10 Codes	Definition
Atrial fibrillation	427.31	I48	Outpatient department records ≥ 3
**Baseline comorbidities**			
Peripheral vascular disease	093.0, 437.3, 440.x, 441.x, 443.1–443.9, 47.1, 557.1, 557.9, V43.4	I70.x, I71.x, I73.1, I73.8, I73.9, I77.1, I79.0, I79.2, K55.1, K55.8, K55.9, Z95.8, Z95.9	Outpatient department records ≥ 3
Systemic embolism	444	I74.01–74.09	Outpatient department records ≥ 3
Hypertension	401, 402	I10–I16	Outpatient department records ≥ 3
Hyperlipidemia	272	E78	Outpatient department records ≥ 3
Diabetes mellitus	250	E10.0, E10.1, E10.9, E11.0, E11.1, E11.9	Outpatient department records ≥ 3
Chronic liver disease	570, 571, 572	B150, B160, B162, B190, K704, K72, K766, I85	Outpatient department records ≥ 3
Ischemic stroke	433, 434, 436	I63, I64	Outpatient department records ≥ 3
Chronic kidney disease	580–589	I12, I13, N00, N01, N02, N03, N04, N05, N07, N11, N14, N17, N18, N19, Q61	Outpatient department records ≥ 3
Chronic lung disease	490, 491.0, 491.1, 491.20– 491.22, 491.8, 491.9, 492.0, 492.8, 493.00–493.02 493.10–493.12, 493.20–493.22, 493.81, 493.82, 493.90–493.92, 494.0, 494.1, 495.8, 495.9, 496, 500, 502, 503, 504, 505, A323, A325	J44	Outpatient department records ≥ 3
Congestive heart failure	428	I11.0, I13.0, I13.2, I42.0, I50, I50.1, I50.9	Outpatient department records ≥ 3
Transient ischemic attack	435	G45	Outpatient department records ≥ 3
Percutaneous coronary intervention	procedure codes of the Taiwan NHI: 33076A, 33076B, 33077A, 33077B, 33078A, 33078B		Discharge
Malignancy	140.0–208.9	C	Outpatient department records ≥ 2
**Clinical outcomes**			
New ischemic stroke (ischemic stroke, transient ischemic attack)	433, 434, 436 435	I63, I64 G45	Discharge
Myocardial infarction	410, 411, 412	I21–I25	Discharge
Bleeding (intracranial hemorrhage, gastrointestinal and other critical site bleeding	430, 431, 432, 852, 853 456.0, 456.2, 455.2, 455.5, 455.8, 530.7, 530.82, 531.0–531.6, 532.0–532.6, 533.0–533.6, 534.0–534.6, 535.0–535.6 537.83, 562.02, 562.03, 562.12 562.13 568.81, 569.3, 569.85, 578.0, 578.1, 578.9 423,0, 459.0, 568.81, 593.81, 599.7, 623.8, 626.32, 626.6, 719.1, 784.7, 784.8, 786.3	I60, I61, I62 K250, K260, K270, K280, K290 D62, J942, H113, H356, H431, N02, N95, R04, R31, R58	Discharge

### Statistical analysis

This research used the Chi-square test for categorical variables (shown as numbers and percentages; N %) and analysis of variance for continuous variables (shown as mean and standardized differences; mean ± SD) to analyze three different medication groups according to baseline demographics. We used 1:1 ratio propensity score matching to reduce the effect of confounding factors. The variables included age, sex, CHA2DS2-VASc scores, and history of disease (Table 1). The Kaplan–Meier method was used to evaluate time-to-death outcomes from the index date and the Cox proportional hazard regression model to estimate the hazard ratios (HR), 95% confidence interval (CIs) for mortality rates up to 3 years. We used SAS 9.4 software (SAS Institute, Cary, NC, USA). In the two-tailed tests, *P* < 0.05 indicated statistical significance.

### Ethics approval

The requirement for informed consent was waived by the Fu Jen Catholic University Institutional Review Board (C108121) in Taiwan because due to the retrospective nature of the study and all NHIRD data had been de-identified.

## Results

**Figure 2:**
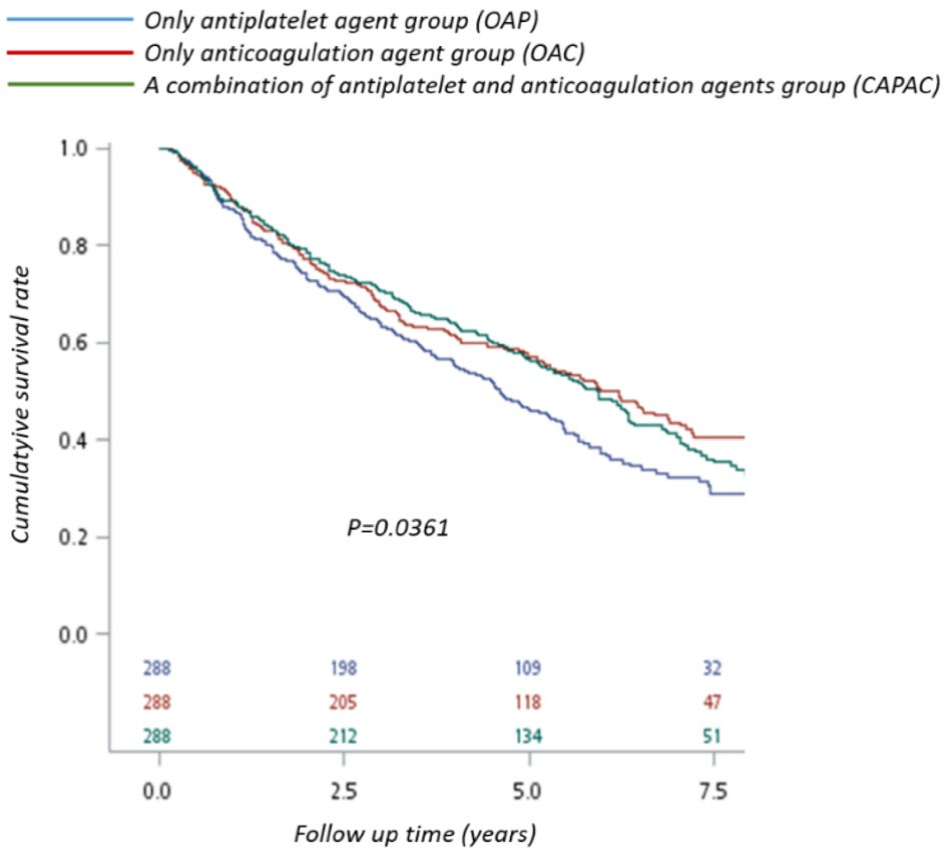
Kaplan–Meier analysis for cumulative survival rates, stratified by different medicine therapy groups. The OAP group showed a higher mortality rate than the CAPAC and the OAC groups in the acute stage and after the follow-up.

After exclusions, we enrolled 2116 consecutive NVAF patients who received successful CAS with regular follow-up between January 1, 2010, and December 31, 2016. All patients received bare-metal stents due to the health insurance regulations of Taiwan. A total of 587, 477, 49, and 304 patients took OAP, OAC, DAP, and CAPAC with persistent drug adherence. The data showed a diversity of drug choice, which suggested difficulty in drug selection. Moreover, around one-third of all patients (n = 733) had inconsistent treatment records. The leading causes of mortality were vascular events, accounting for more than one-third of all deaths. Patients who took OAP or OAC were older and had relatively higher CHA2DS2-VASc scores than patients who took CAPAC (Table 2). Moreover, there was a higher proportion of comorbidities like DM, CKD, and COPD in the OAP group patients. The final analysis was based on cross-comparison after matching between groups except the DAP group because there were not enough patients who took dual antiplatelet agents consistently after CAS to match with other groups. For limited sample size, we could not balance all variables equally in these subgroups. Our final selection is matching different therapy groups mainly according to age, sex, and CHA2DS2-VASc scores to compare primary outcomes. The CHA2DS2-VASc score that includes most comorbidities is an excellent evaluation tool for the risk of stroke in patients with NVAF^25^. It is also effective in predicting mortality rate of patients with AF or heart failure, mortality rate after episodes of ischemic stroke, and the clinical outcomes of patients undergoing CAS^26–30^. The OAP group showed a higher mortality rate in the acute stage and at the end of follow-up compared with the CAPAC and OAC groups (Table 3 and Fig. 2). Administration of an anticoagulation agent seemed to be an essential protective factor influencing 3-year mortality with a hazard ratio of 0.624 (Table 4). There was no significant difference between these three groups in major bleeding, ischemic stroke, or vascular events in the acute stage. However, there was a trend toward fewer vascular events, especially ischemic stroke, but more bleedings, observed in the CAPAC group.

**Table 2:** Baseline characteristics of non-valvular atrial fibrillation patients who had carotid angioplasty and stent placement (CAS), according to treatment type.

Variables		Before matching						
		Only antiplatelet agent (N = 587)		Only anticoagulation agent (N = 477)		A combination of antiplatelet and anticoagulation agents (N = 304)		*p*-value
		n	%	n	%	N	%	
Gender	Female	224	38.16	232	48.64	119	39.14	0.0014
	Male	363	61.84	245	51.36	185	60.86	
Age, mean (SD) year		73.79 (9.29)		73.13(10.22)		71.63 (11.03)		0.008
Total cost (NT$1000)		172,839 (148,860)		188,872 (202.553)		190,859 (165,104)		0.2873
CHA2DS2-VASc score		4.261 (1.613)		4.092 (1.690)		3.783 (1.710)		0.0002
**Baseline (previous history)**								
PVD		267	45.49	181	37.95	112	36.84	0.0117
Systemic embolism		114	19.42	113	23.69	69	22.7	0.2136
Hypertension		209	35.6	157	32.91	97	31.91	0.4710
Hyperlipidemia		365	62.18	257	53.88	156	51.32	0.0021
Diabetes mellitus		346	58.94	211	44.23	124	40.79	<0.001
Liver cirrhosis		23	3.92	16	3.35	10	3.29	0.8441
Stroke		313	53.32	270	56.60	161	52.96	0.4813
CKD		217	36.97	113	23.69	64	21.05	<0.001
COPD		309	52.64	237	49.69	136	44.74	<0.001
CHF		381	64.91	296	62.05	190	62.5	0.5911
TIA		313	53.32	272	57.02	161	52.96	0.3979
PCI		42	7.16	13	3.19	12	3.95	0.0027

NOAC, Non-vitamin K antagonist oral anticoagulant; NT$, New Taiwan dollar; SD, standard deviation; CHA2DS2-VASc, congestive heart failure, hypertension, age 75 years or older, diabetes mellitus, previous stroke/transient ischemic attack, vascular disease, age 65 to 74 years, female; PVD, peripheral vascular disease; CKD, chronic kidney disease; COPD, chronic obstructive pulmonary disease; CHF, congestive heart failure; TIA, transient ischemic attack; PCI, percutaneous coronary intervention.

**Table 3:** Clinical outcomes of different therapeutic groups after matching by age, sex, CHA2DS2-VASc score and baseline comorbidities.

Variables		After matching												
		Only antiplatelet agent (N = 288)				Only anticoagulant group (N = 288)				A combination of antiplatelet and anticoagulation agents (N = 288)				*p*-value
		n		%		n		%		n		%		
Gender	Female	120		41.67		120		41.67		116		40.28		0.9264
	Male	168		58.33		168		58.33		172		59.72		
Age group	40–65	75		26.04		64		22.22		74		25.69		0.5008
	≥ 65	213		73.96		224		77.78		214		74.31		
Age, mean (SD), y		71.74(10.18)				72.43(10.14)				71.89(10.82)				0.7053
Total cost (NT$1000)		157,808(130,735)				186,524(199,706)				189,469(165,872)				0.0443
CHA2DS2-VASc score		3.861(1.619)				3.861(1.619)				3.861(1.619)				1
**Baseline (Previous history)**														
PVD		119	41.32		106		36.81		107		37.15		0.4639	
Systemic embolism		56	19.44		66		22.92		67		23.26		0.4715	
Hypertension		87	30.21		88		30.56		96		33.33		0.6754	
Hyperlipidemia		172	59.72		149		51.74		148		51.39		0.0758	
Diabetes mellitus		156	54.17		126		43.75		121		42.01		0.0067	
Liver cirrhosis		13	4.51		12		4.17		10		3.47		0.8118	
Stroke		132	45.83		159		55.21		155		53.82		0.0522	
CKD		117	40.63		65		22.57		61		21.18		< 0.001	
COPD		136	47.22		141		48.96		129		44.79		0.6026	
CHF		172	59.72		172		59.72		183		63.54		0.5551	
TIA		132	45.83		160		55.56		155		53.82		0.045	
PCI		23	7.99		11		3.82		11		3.82		0.034	
**Outcome**														
Mortality (0–1Y) (n)		36	12.50		31		10.76		31		10.76		0.7500	
Mortality (0–2Y) (n)		77	26.74		66		22.92		61		21.18		< 0.001	
Mortality (0–3Y) (n)		103	35.76		92		31.94		84		29.17		0.2357	
Mortality during whole follow-up		187	64.93		155		53.82		176		61.11		0.0219	
New ischemic stroke		18	6.25		19		6.6		11		3.82		0.2844	
Myocardial infarction		11	3.82		12		4.17		12		4.17		0.9707	
Vascular events		29	10.07		31		10.76		23		7.99		0.5	
Bleeding		103	35.7		117		40.6		129		44.8		0.087	

NOAC, Non-vitamin K antagonist oral anticoagulant; NT$, New Taiwan dollar; SD, standard deviation; CHA2DS2-VASc, congestive heart failure, hypertension, age 75 years or older, diabetes mellitus, previous stroke/transient ischemic attack, vascular disease, age 65 to 74 years, female; PVD, peripheral vascular disease; CKD, chronic kidney disease; COPD, chronic obstructive pulmonary disease; CHF, congestive heart failure; TIA, transient ischemic attack; PCI, percutaneous coronary intervention.

**Table 4:** Cox proportional model to estimate the hazard ratios of 3-year mortality.

Variables		Adjusted HR (95% CI)	*p*-value
**Gender**			
Female	Ref	1	
Male		0.893 (0.683–1.166)	0.4042
**Age**			
40–65	Ref	1	
≥ 65		1.246 (0.875–1.773)	0.2223
**CHA2DS2-VASc score**			
< 4	Ref	1	
≥ 4		1.217 (0.841–1.761)	0.2982
**Group**			
Only antiplatelet agent	Ref	1	
Only anticoagulation agent		0.624 (0.459–0.849)	0.0096
A combination of antiplatelet and anticoagulation agents		0.846(0.622–1.151)	0.2869
COPD	(ref: No)	1.213(0.938–1.568)	0.1402
CKD		0.887(0.687–1.146)	0.358
CHF		1.122(0.823–1.529)	0.4675
Diabetes		1.295(0.986–1.702)	0.0633
Hypertension		0.748(0.57–0.983)	0.0376
Hyperlipidemia		0.904(0.697–1.172)	0.4443
Stroke		1.143(0.869–1.504)	0.3395
Ischemic heart		0.864(0.544–1.374)	0.5379

CHA2DS2-VASc: congestive heart failure, hypertension, age 75 years or older, diabetes mellitus, previous stroke/transient ischemic attack, vascular disease, age 65 to 74 years, female; CKD: chronic kidney disease; COPD: chronic obstructive pulmonary disease; CHF: congestive heart failure.

## Discussion

In-stent restenosis and cardioembolic stroke should be prevented in patients with NVAF who receive CAS for symptomatic high-grade carotid stenosis. Even after CAS, patients with a history of severe carotid atherosclerosis have a high risk of composite vascular events such as MI and vascular death. The combination of antiplatelet and anticoagulation agents might be a reasonable choice for these patients after intervention for the coexisting risks of atherosclerotic and cardioembolic events in these patients. There has been much discussion about combining antiplatelet and anticoagulation agents for AF and thrombotic events in cardiovascular disease^20–23,31^. According to recent cohort studies, for patients with AF after acute coronary syndrome or PCI, triple therapy with dual antiplatelet and one anticoagulation agent should be used briefly, with caution. Then changing to treatment with one NOAC and P2Y12 inhibitor is recommended^19^. Cardiovascular and cerebrovascular diseases have a similar etiology and pathophysiology. However, a recent study investigating combination therapy for stable peripheral or carotid artery disease showed the benefit of reducing major adverse limb events at the cost of increasing major bleeding^32^. There are no guidelines or extensive cohort studies providing evidence on whether to follow the same track in managing patients with coexisting AF and carotid artery stenosis.

To our knowledge, there is no large randomized trial focusing on antiplatelet and anticoagulation agents after CAS in patients with underlying NVAF. Although there are several articles debating this issue, we are the first retrospective national study with convincible sample size. According to our NHIRD 2008–2017 data, Taiwanese patients with coexisting AF and symptomatic carotid stenosis had a high mortality rate after CAS, and less than half were alive after five years. Vascular events were the primary cause of death even with intensive regular medical prevention. The lack of a standard treatment is shown by the divergent drug selection in our data.

Several periprocedural complications, such as hyperperfusion syndrome, gastrointestinal hemorrhage, MI, systemic embolism, stroke, and TIA, occurred frequently within 30 days of CAS with an incidence of around 4%^33^. These events during admission for CAS may be difficult to determine from our data and might have influenced drug choice. Our study focused on the drug choice for patients with NVAF within half year after CAS. In comparing primary outcomes, a combination of antiplatelet and anticoagulation agents or only anticoagulation agents in the acute stage seemed more helpful for these patients than only an antiplatelet agent. A lower incidence of vascular events, especially ischemic stroke, was observed in the combination group. There was an increased risk of bleeding, which was not statistically significant. A strategy focusing on anticoagulation effects plays a vital role in this situation. Compared to the previous study focusing on antithrombotic agents after acute coronary syndrome or PCI in AF, our analysis showed fewer vascular events but more bleeding^22^. As we previously mentioned, most in-stent restenosis and vascular events happened within the first month in patients without NVAF receiving CAS who would not be included in our study. Further, our study was based on observational data according to the admission ICD codes, therefore, some minor strokes or vascular events without admission might have been missed. However, overall mortality rates may reflect the sum of these physical problems. Treatments containing anticoagulation agents must be implemented in clinical practice in patients with NVAF receiving CAS. A combination of a single antiplatelet and an anticoagulation agent might be indicated when there are concerns about possible vascular events. It might be beneficial to initially prescribe combination therapy during the high-risk period. Kouhei also suggested administering one NOAC plus a single antiplatelet agent in a published case series^34^. Clopidogrel may be a good choice of antiplatelet agent according to studies of PCI^22,35^. Our study did not separate patients taking aspirin and warfarin or clopidogrel and NOACs, which might be the reason more bleeding was recorded.

Current European guidelines recommend lifelong OAC with no antiplatelet agent beyond one year after coronary stenting in patients with NVAF^36^. Anticoagulation monotherapy was superior to aspirin or clopidogrel alone in preventing ischemic events. An anticoagulant plus a single antiplatelet agent did not improve protection but increased the risk of bleeding after the acute stage of MI. Our study showed that a single anticoagulation agent provided better prevention than a single antiplatelet agent and had a lower bleeding risk than combination therapy. Prevention of vascular events and bleeding risk resulted in low mortality during follow-up, and most hazard ratio reduction was recorded in the only anticoagulation therapy group. One anticoagulant seemed to be enough for most cases with NVAf after CAS.

Based on the experience of previous clinical trials, the use of dual antiplatelet drugs for a short period is the consensus for medical management after CAS^37^. But these trials were non-blinded and had small numbers^38^. The appropriate duration of dual antiplatelet therapy after CAS is unknown. In line with the existing guidelines for the period covered by our study, dual antiplatelet agents were prescribed for one month after intervention. Therefore, in our study, most physicians stopped dual antiplatelet agents after 1 month, and there were only 49 cases that were prescribed dual antiplatelet agents consistently in the half-year following carotid stenting. According to experience from studies focusing on acute coronary syndrome or PCI in AF, triple therapy with dual antiplatelet agents and one anticoagulation agent is unnecessary. It increases bleeding risk one month after the intervention for most cases. These two kinds of medicine selection were not considered in our study.

This study has several limitations. It is only a retrospective observational study based on NHIRD data. The identification of comorbidities and complications is based on diagnostic codes listed in the database; some coding errors may have occurred. A diagnostic bias concerning the endpoints can be ruled out due to specific operation codes and ICD codes for significant events being checked carefully during admission according to health insurance requirements. The disease severity and any underlying diseases may affect the physician’s drug choice. To reduce the observational study’s inherent bias, we matched patients according to age, CHA2DS2-VASc score, and comorbidities before analysis where possible. There are no clear post-CAS drug treatment guidelines, especially for patients with NVAF. Prescribing may vary and, at times, were changed due to the clinical physicians’ choice. We decided to group the patients according to drug use information obtained from the outpatient pharmacy prescription database. Only patients who took one kind of medicine continuously for at least half a year were enrolled. However, drug compliance and the influence of lifestyle factors such as smoking, overeating, and poor sleep quality could not be accounted for in this study.

In summary, this study may offer preliminary evidence for clinical practice and insights for further investigation. One anticoagulation agent had a lower mortality rate and relatively low bleeding risk. The Cox proportional model estimate of the HR showed that drug choice of one anticoagulation agent might be the critical factor influencing mortality at 3-year follow-up. One anticoagulation agent may be standard medical therapy after CAS in patients with NVAF. Combination therapy with one antiplatelet and one anticoagulation agent may be considered as initial therapy when there are concerns about possible vascular events. NOACs might be a better choice than warfarin, according to some studies in Asian populations^39–41^. Clopidogrel may be a good choice of antiplatelet agent.

Prompt intervention followed by intensive medical control with anticoagulation therapy is the most reasonable choice for patients with NVAF and high-grade carotid stenosis. Further large-scale randomized trials are needed to confirm this.
